# Osseointegration of Titanium Implants in a Botox-Induced Muscle Paralysis Rat Model Is Sensitive to Surface Topography and Semaphorin 3A Treatment

**DOI:** 10.3390/biomimetics8010093

**Published:** 2023-02-25

**Authors:** Jingyao Deng, D. Joshua Cohen, Michael B. Berger, Eleanor L. Sabalewski, Michael J. McClure, Barbara D. Boyan, Zvi Schwartz

**Affiliations:** 1 Department of Biomedical Engineering, College of Engineering, Virginia Commonwealth University, Richmond, VA 23284, USA; 2 VCU DaVinci Center for Innovation, Virginia Commonwealth University, Richmond, VA 23284, USA; 3 Wallace H. Coulter Department of Biomedical Engineering, Georgia Institute of Technology, Atlanta, GA 30332, USA; 4 Department of Periodontics, University of Texas Health Science Center at San Antonio, San Antonio, TX 78229, USA

**Keywords:** titanium implants, surface topography, biomimetic multiscale micro/nano texture, osseointegration, botox, semaphorin 3A, in vivo bone phenotype

## Abstract

Reduced skeletal loading associated with many conditions, such as neuromuscular injuries, can lead to bone fragility and may threaten the success of implant therapy. Our group has developed a botulinum toxin A (botox) injection model to imitate disease-reduced skeletal loading and reported that botox dramatically impaired the bone formation and osseointegration of titanium implants. Semaphorin 3A (sema3A) is an osteoprotective factor that increases bone formation and inhibits bone resorption, indicating its potential therapeutic role in improving osseointegration in vivo. We first evaluated the sema3A effect on whole bone morphology following botox injections by delivering sema3A via injection. We then evaluated the sema3A effect on the osseointegration of titanium implants with two different surface topographies by delivering sema3A to cortical bone defect sites prepared for implant insertion and above the implants after insertion using a copper-free click hydrogel that polymerizes rapidly in situ. Implants had hydrophobic smooth surfaces (PT) or multiscale biomimetic micro/nano topography (SLAnano). Sema3A rescued the botox-impaired bone formation. Furthermore, biomimetic Ti implants improved the bone-to-implant contact (BIC) and mechanical properties of the integrated bone in the botox-treated rats, which sema3A enhanced. This study demonstrated the value of biomimetic approaches combining multiscale topography and biologics in improving the clinical outcomes of implant therapy.

## 1. Introduction

The success rate of dental, spinal, or orthopedic implants has always been challenging for compromised patients, such as the elderly, smokers, diabetics, and osteoporosis [1,2]. To increase implant success, research on implants with modifications such as complex micro-/nano-topography, wettability, and altered surface chemistry has been ongoing for many years [3,4,5].

Titanium and its alloys are the most common materials for implant therapy in the bone due to their superior corrosion resistance, easy processing, mechanical support properties, and biocompatibility [6,7]. The integration of an implant with surrounding bone is a highly structured biological process consisting of protein adsorption, immune cell modulation, mesenchymal cell recruitment and osteoblast differentiation, primary bone formation and vascularization, bone remodeling, and mature bone formation [4]. This process, termed osseointegration, is defined operationally as the degree of bone-to-implant contact (BIC) [4].

Many studies aim to create implant surfaces that mimic the physical structure of bone surfaces following resorption by osteoclasts [8,9]. The unique structure of the resorbed bone surface during bone remodeling, including micro-scale, submicron-scale, and nano-scale features, favors subsequent bone formation, and implant surfaces that possess these features exhibit enhanced osseointegration and long-term implant success [4,5,10,11]. This has been demonstrated in rodent models with compromised bone quality, including osteoporosis and diabetes [12,13,14,15].

Peri-implant bone formation depends on the ability of bone marrow stromal cells (MSCs) to colonize implant surfaces and differentiate into osteoblasts [4]. Osteoprogenitor cells exhibit osteoblast differentiation markers and produce local factors in a well-orchestrated manner when responding to the biomimetic implant surface properties that are characterized by physical features similar to the surface of osteoclast-resorbed bone [16]. This results in a microenvironment favoring osteogenesis [17,18], anti-inflammatory tissue regeneration [19,20,21], and new blood vessel formation [22,23], as well as modulating osteoclast resorptive activities [24].

This osteogenic microenvironment is dynamic and underlying mechanisms are not fully understood. However, studies using animal models indicate that supplementing the normal production of various local factors using an exogenous biologic approach can aid or enhance the surface-mediated osteogenic microenvironment [25,26]. One indicator of the effect of the osteogenic surface on MSC differentiation is the upregulation of osteocalcin (OCN) [3], a marker of a well-differentiated osteoblast. This is partly due to the production of the osteoinductive protein, bone morphogenetic protein 2 (BMP2) [27].

In addition to BMP2, MSCs respond to biomimetic Ti surface features with the increased production of a nerve-derived factor, semaphorin 3A (sema3A) [28]. Sema3A, originally identified as an axon guidance molecule, is osteoprotective [29,30,31,32]. It can increase osteoblastic differentiation while regulating osteoclast resorptive activities [32]. The treatment of MSCs cultured on Ti disks with a grit-blasted, acid-etched surface with sema3A further enhanced the effect of the biomimetic topography on osteoblastic differentiation, including the production of OCN and osteoprotegerin (OPG) [28]. These observations suggest that sema3A may function similarly in vivo.

Several studies have shown that Ti implants with a biomimetic surface topography result in improved osseointegration in animal models that exhibit a compromised bone quality compared to implants with a smooth topography created by machining [33,34,35,36]. Most of these studies have used rats or mice with an induced diabetic phenotype or an osteoporotic phenotype resulting from ovariectomy [12,13,33,34,35,36]. A compromised bone quality can also result from disuse or mechanical unloading situations, which can be caused by long-term bed rest, neuromuscular injuries, space flight, or spinal cord injuries [15,37,38,39]. To assess whether Ti implant surface features would improve osseointegration under conditions where the bone was mechanically unloaded, we established a rat model using the neurotoxin botulinum toxin A (botox), which inhibits the release of acetylcholine from the neuromuscular junctions, causing paralysis. We found that osseointegration was drastically inhibited in the absence of mechanical stress [40].

We previously showed that the local delivery of human recombinant sema3A improved the osseointegration of Ti implants with a biomimetic surface topography placed transcortically in diabetic rat femurs, which have a similar bone phenotype to mechanically unloaded bone with respect to the loss of trabeculae in the metaphysis [40,41]. This suggested that sema3A might also improve implant integration in mechanically unloaded bone. Accordingly, we used the botox injection model to evaluate the effectiveness of biomimetic surface topography in the absence of mechanical loading and to assess if the local delivery of the nerve-derived factor sema3A would improve osseointegration to the level found in healthy animals. The study determined whether sema3A treatment is sufficient to stimulate osseointegration with a smooth Ti implant to the levels observed when using a Ti surface with a multiscale biomimetic topography. We then assessed if the addition of sema3A is able to enhance the osseointegration of a Ti implant with a biomimetic surface in the botox-compromised model.

## 2. Materials and Methods

### 2.1. Implant Manufacturing

Titanium Implants machined from grade 4 titanium rods to be 2.5 mm in diameter, 3.5 mm in length, and 0.8 mm in pitch were customized to fit in a rat femur by Institut Straumann AG (Basel, Switzerland). The machined implants were designated “pre-treatment” (PT). The PT implants were blasted with 250–500 μm Al_2_OH_3_ grit and acid-etched in a mixture of HCl and H_2_SO_4_, resulting in a complex microrough topography (SLA), and then processed under nitrogen and stored in 0.9% sterile saline, resulting in a hydrophilic surface that had nanoscale features hydrophilic (modSLA). PT and modSLA implants were sterilized using γ irradiation. In order to obtain hydrophobic implants that had both the SLA microroughness and the added nanoscale features found on the modSLA surfaces, the modSLA implants were removed from the sterile saline package in a biological safety cabinet under sterile conditions and aged for at least 1 month to generate the SLAnano surfaces, which were repackaged in aluminum foil. The physical and chemical properties of the PT and SLAnano surfaces have been described in detail [42].

We previously reported that the osseointegration of modSLA implants, which have a hydrophilic surface, is impaired in the botox-compromised rat model [40]. In the present study, our goal was to focus on surface topography without the confounding variable of wettability, and the PT and SLAnano surfaces are both hydrophobic. Thus, the present experimental design enabled us to focus on the contribution of biomimetic multiscale topography on osseointegration in this mechanical unloading model and to assess the contribution of sema3A to the process.

### 2.2. Hydrogel Preparation

We used a copper-free click hydrogel as the sema3A delivery vehicle. We have successfully used click hydrogels to deliver biologics, drugs, and antibodies to bone defect sites, with no evidence of toxicity [41,43,44]. Swelling is minimal, and following an initial burst release, the payload is released at a steady rate, with degradation occurring over a 14-day period [41,43,44]. The techniques used to prepare the hydrogel were adapted from previous work [41,43,44]. Briefly, the copper-free click-based chemistry was used to combine two aqueous solutions that underwent in situ chemical crosslinking to create the quickly polymerizing hydrogel. A thiol-Michel addition reaction involving PEG-dithiol and dibenzocyclooctyne maleimide (DBCO-maleimide) was used to create a poly-ethylene glycol (PEG) crosslinker that has been functionalized with dibenzocyclooctyne (DBCO). When combined with an azide-functionalized acylate polymer, the DBCO-functionalized precursor created an in situ crosslinked hydrogel. Reversible addition-fragmentation chain transfer (RAFT) polymerization, which allows for the precise control of azide functionality, was used to create PEG-N3 from azide functionalized and non-functionalized PEG methacrylate monomers to produce a water-soluble, non-fouling multivalent azide functionalized polymer. The components were synthesized at a commercial facility under Good Laboratory Practice controls (Syngene International Limited, Bangalore, India) according to our requirements and shipped lyophilized to our laboratory. Before use, the components were stored at −80 °C after being reconstituted in sterile 1X PBS (ThermoFisher Scientific, Waltham, MA, USA). The hydrogels were formed by combining PEG-N3 (50%; w:v) and PEG-DBCO (12.5%; w:v) at a 1:2 (v/v) ratio.

### 2.3. Animals and Surgical Procedures

The Institutional Animal Care and Use Committee at Virginia Commonwealth University approved all the animal procedures. The National Institutes of Health’s guide for the care and use of laboratory animals was followed in all experiments. Animals were kept in an AALAC-approved animal facility in indoor housing with a 12h/12h light/dark cycle and individually ventilated, solid-bottomed polysulfone cages that allow for temperature and humidity adjustment within ranges appropriate for the animals.

For all animal procedures, 5% isoflurane gas with O_2_ was used to induce anesthesia and kept at 2.5% after. Animals recovered consciousness on a water-circulating warming pad before returning to the vivarium. For surgical procedures, 1 mg/kg of sustained-release buprenorphine (ZooPharm, Windsor, CO, USA) was administered pre-operatively and subcutaneously to provide a minimum of 72 h of postoperative analgesia.

#### 2.3.1. Therapeutic Effect of Sema3a on the Botox-Induced Compromised Bone Phenotype

**Figure 1:**
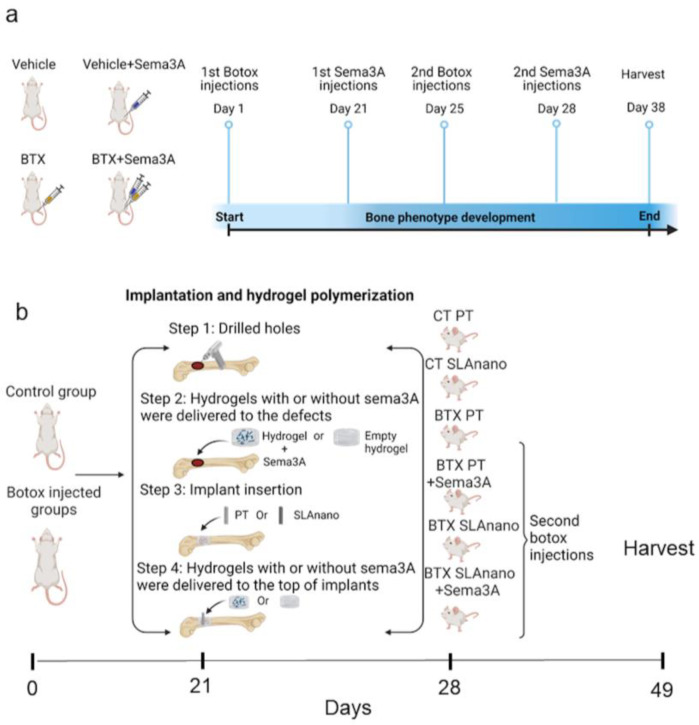
Schematic of the experimental procedures. (**a**) Sprague Dawley rats were divided into 4 groups: BTX and BTX+Sema3A groups underwent botox injection on day 1 and day 25 to ensure the botox effect for the entire study. Veh+Sema3A and BTX+Sema3A groups had sema3A injections on day 21 and day 28. Veh group and BTX group were injected with sterile saline as vehicle controls. Rats were harvested on day 38. (**b**) Sprague Dawley rats were divided into 6 groups. On day 1, groups 3–6 received 8 units of botulinum toxin type A (BTX) with 2 units to the paraspinal muscles, upper and lower quadriceps, hamstring, and calf. On day 21, groups 1, 3, and 4 received PT implants (smooth), and 2, 5, and 6 received SLAnano implants screwed into the right distal femurs. Groups 4 and 6 were treated with recombinant sema3A delivered via hydrogel in the drilled bone marrow cavity before implant insertion and above the implants after implant insertion. On day 28, botox groups received a second injection of BTX. On day 49, all rats were sacrificed, and femurs were harvested for microCT scanning and removal torque mechanical testing.

In total, 29 male Sprague Dawley rats (SD) weighing 300–325 g (Charles River Laboratories, Wilmington, MA, USA) were randomly divided into 4 groups: control (veh, n = 4), control with sema3A injections (veh+sema3A, n = 8), botox injections (BTX, n = 8), and botox injections with sema3A injections (BTX+sema3A, n = 9). One extra rat was prepared and randomly assigned to the BTX+sema3A group. Botulinum toxin type A (onabotulinumtoxinA; BOTOX®, Allergan, Inc. Irvine, CA, USA [botox]) was dissolved in 0.9% saline (10 units/mL) [40]. On day 1 and day 25, for the BTX and BTX+sema3A groups, the right hindlimbs were injected intramuscularly with a total of 8 units of botox distributed as 2 units into the following locations: paraspinal muscles, quadriceps, the hamstrings, and the calf muscles. The contralateral legs were the internal controls (Figure 1a). On day 21 and day 28, recombinant human sema3A was reconstituted in 0.9% sterile saline (100 μg/mL, R&D Systems, Minneapolis, MN, USA), and 6 μg of sema3A (100 μg/mL in 60 μL) were injected into the periosteal layer of the distal end of the third trochanter on the right femurs for veh+sema3A and BTX+sema3A groups, and the same amount of 0.9% sterile saline was injected to the rest of the groups (Figure 1a). On day 38, the rats were humanely euthanized by CO_2_ inhalation and cervical dislocation. Femurs were harvested in 1XPBS and further evaluated by microCT and 3-point bending fracture analysis, which is described in Section 2.4.2.

We opted to use recombinant human sema3A instead of recombinant rat sema3A for two reasons. We knew that human sema3A could enhance surface-mediated osteoblast differentiation of human MSCs in vitro, as well as the production of factors associated with osteogenesis. Moreover, human sema3A restored the osseointegration of Ti implants in a type 2 diabetic rat model to normal levels, demonstrating that it was bioactive in vivo [28,41]. Therefore, the concentrations of the same human recombinant sema3A were adopted for this study.

#### 2.3.2. Effect of Surface Topography on Response to Sema3A

In total, 49 male SD rats weighing 300–325 g (Charles River) were randomly divided into 6 groups: control rats with PT implants (Control+PT, n = 8), control rats with SLAnano implants (Control+SLAnano, n = 8), botox-injected rats with PT implants (BTX+PT, n = 8), botox-injected rats with PT implants and sema3A injections (BTX+PT+sema3A, n = 8), botox-injected rats with SLAnano implants (BTX+SLAnano, n = 8), and botox-injected rats with SLAnano implants and sema3A injections (BTX+SLAnano+sema3A, n = 8). One animal from the BTX+SLAnano group was withdrawn from the study as it met the humane endpoint; thus, BTX+SLAnano had n = 7 for the subsequent tissue analysis. On day 1 and day 28, the same dose of botox was injected into the same muscle groups to BTX+PT, BTX+PT+sema3A, BTX+SLAnano, and BTX+SLAnano+sema3A right hindlimbs. On day 21, all rats were prepared for implant insertion and hydrogel loading surgeries by shaving and cleaning the right hindlimbs with 70% ethanol and 2% chlorhexidine. The implant insertion sites were produced by sequentially drilling a defect with increasing diameter dental drill bits (Ø1.0 mm, Ø1.6 mm, Ø2.0 mm, and Ø2.2 mm) to a depth of 3.5 mm in the distal metaphysis of the femur after separating the adjacent muscles and periosteum.

Recombinant human sema3A (R&D Systems) was reconstituted with the PEG-DBCO crosslinker solution. The hydrogels were formed by combining 5.33 μL of PEG-N3 and 10.66 μL of PEG-DBCO with or without 6 μg of sema3A to the designated groups using separate pipettors to pipette two components into the holes simultaneously. This resulted in 6 μg of sema3A/hydrogel. Threaded PT implants or SLA implants were inserted into the holes after gelation. Cover screws were added to cap the implants. Then, the hydrogels were delivered on top of the implants again with or without sema3A with the same techniques (Figure 1b). Rats were recovered from anesthesia on a water-circulating warming pad and weighed weekly. On day 49, all rats were humanely euthanized, and femurs were harvested in 1X PBS for further analysis.

### 2.4. Tissue Analysis

#### 2.4.1. Micro-Computed Tomography

Femurs were isolated and prepared for microCT scanning (SkyScan 1173, Bruker, Kontich, Belgium) within 24 h of harvest without fixation to evaluate the bone phenotype and peri-implant bone growth. For the first study, to evaluate the effect of sema3A on bone morphology, both distal and proximal ends of the femurs were scanned at a resolution of 1120 × 1120 pixels (isotropic voxel size of 15.82 μm) using a 1.0 mm aluminum filter, at an exposure of 250 ms, with scanning energies of 85 kV and 94 μA [40]. A standard Feldkamp reconstruction was conducted by NRecon Software (Bruker) with a beam hardening correction of 20%, and no smoothing was applied. The quantitative trabecular morphometric parameters, including bone volume/total volume (BV/TV), trabecular number, trabecular thickness, and total porosity, were evaluated for the first animal study. The quantitative cortical morphometric parameters were determined, including BV/TV, the total porosity, and cortical thickness.

For the second animal study, bone-to-implant contact (BIC) was evaluated for each implant by scanning the metaphysis of the distal femurs using a 0.25 mm brass filter, at an exposure of 420 ms, with scanning energies of 120 kV and 66 μA. After reconstruction, total BIC, BIC in the marrow space, and cortical bone BIC were determined using previously described methods [40].

#### 2.4.2. Mechanical Testing

In the first experiment, a 3-point bending study was performed using a BOSE ElectroForce 3200 Series III axis (TA Instruments, New Castle, DE, USA). Bones were positioned so that the femur’s sema3A injection site was in the middle of two support struts that were facing up. The load cell was attached using a triangular prism-pointed testing mount. An axial compressive displacement rate of 0.1 mm per second was used to achieve axial displacement until failure.

In the second experiment, mechanical torque to failure was used to determine the overall implant mechanical integrity using a Bose ElectroForce 3200 Series III Axial-Torsion mechanical testing system equipped with a 445 N/5.7 N m load/torque transducer. Before loading the samples, the load cell was zeroed, and a 0.1 Hz filter was used to cancel the background noise. Femurs were mounted on customized polylactic acid holders with polyurethane adhesive as described [15,40]. The implant mount was customized to fit the implant after removing the cover screws on the top. The sides of the bone holder were then clamped between two flat specimen clamps once the implant had been firmly secured to the mount and was perpendicular to the axis of rotation. The implant was subsequently removed from the surrounding bone by rotating the femurs at 0.1°/ s while rising at a pace of 0.8 mm/360° simultaneously.

After zeroing the initial load and displacement or initial torque and rotation radian, the mechanical examination was done by creating load vs. displacement graphs for 3-point bending analyses and torque vs. radian graphs for torsional analyses. SLM-Shape Language (Modeling version 1.14, MATLAB, MathWorks, Natick, MA, USA) fitted the curve to a bilinear model to separate the linear region from the toe region to eliminate the initial gap potentially between the mount and the implant. The curve was then evaluated for the maximum load at failure (N), stiffness (N m), and toughness at failure (millijoules) and normalized to the cross-sectional area calculated in the microCT analysis for each leg 3-point bending analyses. For the implant osseointegration analysis, it was possible to compute the torque at failure (Nm), torsional stiffness (linear region slope, N m/radians), and torsional energy (area under the curve, millijoules) from the torque vs. degree graphs. The information is displayed as the treatment (right leg) over the control (left leg) as published [40].

### 2.5. Statistical Analysis

A power analysis was performed using an alpha of 0.05 and a power of 80% (delta = 5, sigma = 3, m = 1), which revealed that a minimum of n = 7 per group was required for the study to be statistically significant. An in vivo assessment was done between contralateral legs and treatment legs by Wilcoxon matched-pairs signed rank test (α = 0.05) represented by an asterisk (*) using GraphPad Prism (GraphPad, La Jolla, CA, USA), and a one-way analysis of variance to compare between groups with Tukey’s post hoc test using JMP statistical software (SAS Institute Inc., Cary, North Carolina). A two-way ANOVA was used to compare differences among groups with two independent variances for removal of the torque mechanical test analysis using GraphPad Prism.

## 3. Results

### 3.1. Botox Compromised the Trabecular and Cortical Bone Phenotype at the Distal Metaphysis of the Femurs

**Figure 2:**
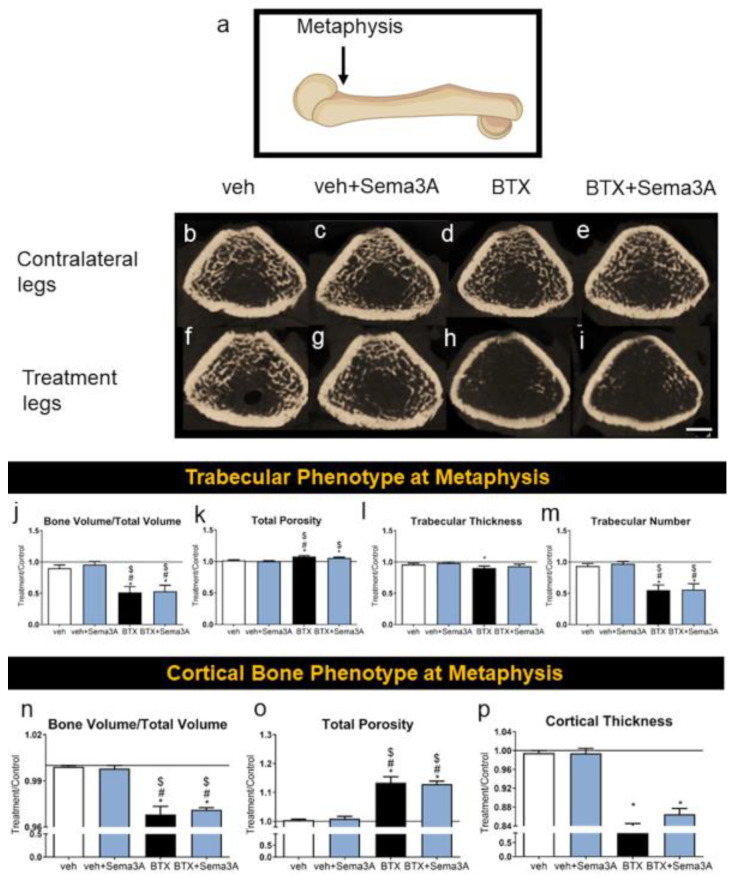
Effect of sema3A on trabecular and cortical bone formation at the distal end of femurs. After 38 days, femurs were isolated and the metaphysis of distal femurs (**a**) was analyzed with 3D microCT reconstructions: vehicle left femur (**b**), vehicle right femur (**f**), veh+Sema3A left femur (**c**), veh+Sema3A right femur (**g**), BTX left femur (**d**), BTX right femur (**h**), BTX+Sema3A left femur (**e**), and BTX+Sema3A right femur (**i**). Trabecular bone volume/total volume (**j**), total porosity (**k**), trabecular thickness (**l**), and trabecular number (**m**) were quantified from the microCT reconstructions. Cortical bone volume/total volume (**n**), total porosity (**o**), and cortical thickness (**p**) were quantified from the microCT reconstructions. Data shown are the means of treatment/contralateral leg for each group ± standard error of n = 5 for the veh group, n = 8 for the veh+Sema3A group, n = 8 for the BTX group, and n = 9 for the BTX+Sema3A group. Comparisons between treatment legs and contralateral legs identified with an asterisk, *, are statistically different at α = 0.05 by the Wilcoxon matched-paired signed rank test. One-way ANOVA was used for comparisons among multiple groups, and Tukey’s post hoc test was used after ANOVA. # *p* < 0.05 vs. veh group, $ *p* < 0.05 vs. veh+Sema3A group. Scale bar = 1 mm.

The trabecular bone and cortical bone phenotype at the distal ends of the femur near the implant insertion site were analyzed by microCT (Figure 2a), and the representative images are shown in Figure 2b–i. The development of a compromised bone phenotype induced by botox injections was demonstrated qualitatively by reduced the trabecular bone formation (Figure 2h) compared to both vehicle groups (Figure 2b,f) and its contralateral leg (Figure 2d). This was further confirmed quantitatively, including a lower BV/TV (Figure 2j), higher total porosity (Figure 2k), and lower trabecular thickness and number (Figure 2l,m). The addition of sema3A did not have any significant effect on increasing the trabecular bone formation in both healthy rats (veh+sema3A, Figure 2j–m) and botox-injected rats (BTX+sema3A, Figure 2j–m). The cortical bone was also affected by a botox injection. Cortical BV/TV was reduced (Figure 2n), the cortical bone total porosity was increased (Figure 2o), and the cortical thickness was reduced (Figure 2p), indicating that botox decreased the cortical bone formation. Additionally, sema3A did not affect the cortical bone formation in healthy or botox-injected rats at the distal metaphysis. The raw data without normalization are also presented in Figure S1.

### 3.2. Sema3A Burst Release Had a Therapeutic Effect on the Botox-Compromised Cortical Bone at Its Injection Sites

**Figure 3:**
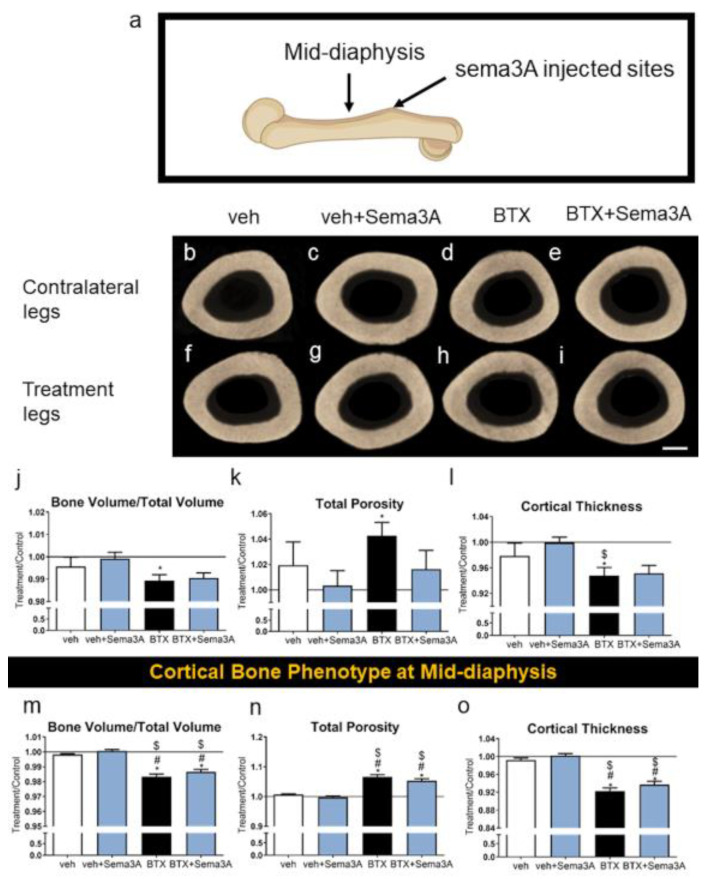
Effect of sema3A on cortical bone formation at the sema3A injected sites. After 38 days, femurs were isolated, and the sema3A injected sites of distal femurs (**a**) were analyzed with 3D microCT reconstructions: vehicle left femur (**b**), vehicle right femur (**f**), veh+Sema3A left femur (**c**), veh+Sema3A right femur (**g**), BTX left femur (**d**), BTX right femur (**h**), BTX+Sema3A left femur (**e**), and BTX+Sema3A right femur (**i**). Cortical bone volume/total volume (**j**), total porosity (**k**), and cortical thickness (**l**) at the sema3A injection sites were quantified from the microCT reconstructions. Cortical bone volume/total volume (**m**), total porosity (**n**), and cortical thickness (**o**) at the mid-diaphysis were quantified from the microCT reconstructions. Data shown are the means of treatment/contralateral leg for each group ± standard error of n=5 for the veh group, n = 8 for the veh+Sema3A group, n = 8 for the BTX group, and n = 9 for BTX+Sema3A group. Comparisons between treatment legs and contralateral legs identified with an asterisk, *, are statistically different at α = 0.05 by the Wilcoxon matched-paired signed rank test. One-way ANOVA was used for comparisons among multiple groups and Tukey’s post hoc test was used after ANOVA. # *p* < 0.05 vs. veh group, $ *p* < 0.05 vs. veh+Sema3A group. Scale bar = 1 mm.

The cortical bone phenotype was evaluated at the sema3A injected sites at the distal side of the third trochanter and at the mid-diaphysis (Figure 3a) and was compared to the contralateral legs. The difference caused by a botox injection on the cortical bones was hard to distinguish in the qualitative images (Figure 3b–i). However, microCT showed that botox reduced BV/TV at the trochanter, and sema3A injections at that site had no effect (Figure 3j). Botox increased the total porosity (Figure 3k) and decreased the cortical thickness (Figure 3l). The Sema3A injections restored the total porosity to normal levels in the botox-treated rats but had no effect on the cortical thickness. Similarly, botox injections reduced BV/TV (Figure 3m), increased the total porosity (Figure 3n), and decreased the cortical thickness (Figure 3o) at the mid diaphysis in comparison to the contralateral legs, and the injection of sema3A had no effect. Overall, botox injections affected the whole bone phenotype by compromising both the trabecular bone and cortical bone formation, and the effect of sema3A on rescuing the compromised bone phenotype was localized and specific to its injection sites. The raw data without normalization are presented in Figure S2.

### 3.3. Biomimetic Surface Topography Improved Osseointegration, and this was Enhanced by Sema3A Treatment

**Figure 4:**
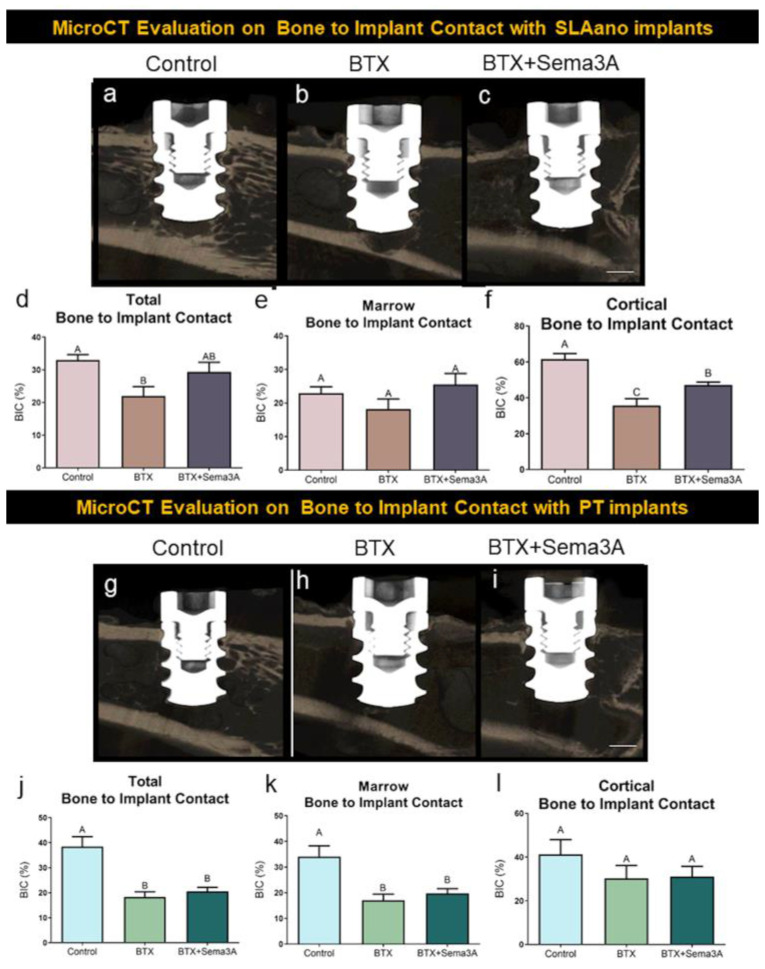
The evaluation of osseointegration by microCT. Male, 12-week-old Sprague Dawley rats were divided into 6 groups: control + PT implants control + SLAnano implants, BTX + PT implants, BTX+ PT + Sema3A, BTX + SLAnano implants, BTX + SLAnano + Sema3A groups. Twnety-one days after botox injection, PT or SLAnano were inserted into the distal end of the right femurs. Sema3A was delivered by hydrogel into the bone marrow space before implant insertions and above implants after insertions. Femurs were harvested after 28 days of osseointegration and prepared for microCT scanning. The representative images from microCT were shown in (**a**) control + SLAnano, (**b**) BTX + SLAnano, (**c**) BTX + SLAnano + Sema3A, and the total bone to implant contact (**d**), bone to implant contact in bone marrow space (**e**), and cortical bone to implant contact (**f**) were quantified from 3D microCT images. The osseointegration of PT implants was also evaluated, and the representative images were shown as (**g**) control + PT, (**h**) BTX + PT, and (**i**) BTX+PT+Sema3A. The total bone to implant contact (**j**), bone implant contact in the marrow space (**k**), and cortical bone to implant contact (**l**) were quantified. Data shown are the means for each group ± standard error of n = 8 for control + PT, and control + SLA, n = 7 for BTX + SLAnano, and n = 8 for BTX + SLAnano + Sema3A, BTX + PT, BTX + PT +Sema3A group. Groups not sharing a letter were significantly different (A vs. B vs. C) by one-way ANOVA and Tukey’s post hoc test (α = 0.05). Groups that share a letter (A vs. A or AB vs. B for example) are not significantly different. Scale bar = 1 mm.

PT and SLAnano implants were inserted into the metaphysis of the distal femurs as described in the methods. The representative images are shown in Figure 4a–c for SLAnano implants. Botox injections caused a reduction in trabecular bone in the bone marrow compartment, regardless of whether the rats were treated with sema3A (Figure 4a–c). Botox reduced the total BIC and cortical BIC compared to the vehicle control groups (Figure 4a,d,f), but botox injections did not affect BIC in the bone marrow compartment (Figure 4e). Even though it was not significantly different, marrow BIC was 18% less in the BTX group than in the control group. The addition of sema3A to the BTX group had a mean of 25% for marrow BIC for SLAnano, which was higher (not statistically significant) than both marrow BIC in the botox (18.22%) and control group (22.95%) (Table S1). The addition of sema3A eliminated the difference in total BIC between the healthy and botox-injected rats, mainly due to significantly higher cortical BIC after a sema3A treatment in the BTX group (Figure 4d,f).

PT implants had qualitatively fewer bone trabeculae associated with them than were present around SLAnano implants (compare Figure 4a,g). The PT implants did not alter the botox-compromised bone phenotype (Figure 4h,i). The total BIC for PT implants in botox-treated animals was significantly lower than in the control groups, and the BIC mainly contributed to the decrease in the bone marrow space. This was different from SLAnano implants, in which the decreased total BIC was mainly contributed by lower cortical BIC in the botox-treated rats. Sema3A did not show any effects on improving BIC for PT implants, but there was a therapeutic effect on improving BIC for SLAnano implants under botox-compromised conditions (Figure 4d,f).

### 3.4. Ti Surfaces with a Multiscale Biomimetic Topography Improve Osseointegration for Mechanical Unloading Situations Regardless of Sema3A Treatment

**Figure 5:**
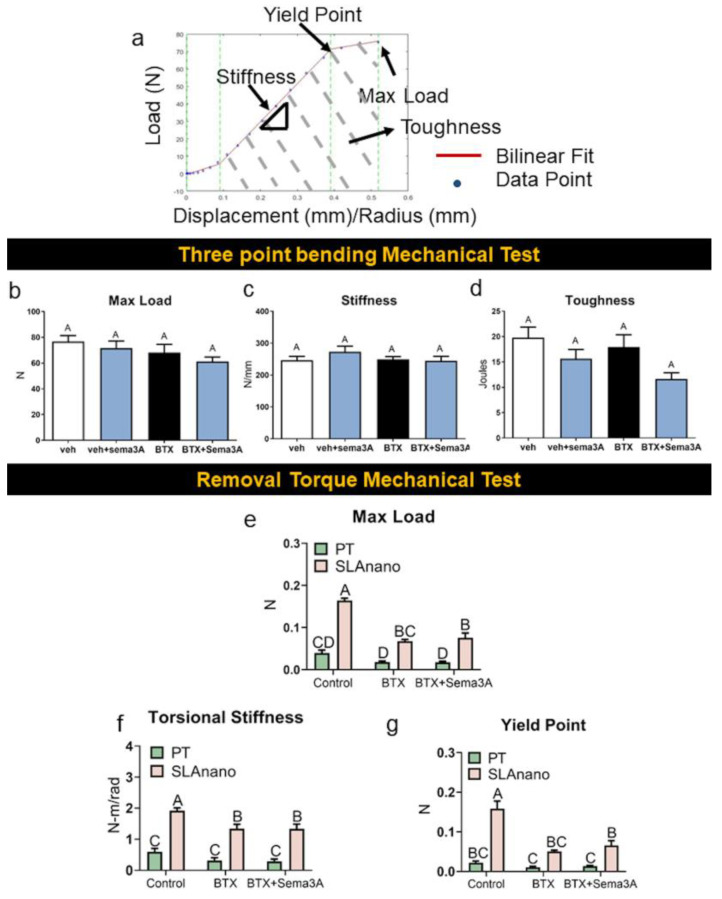
The effect of sema3A on the mechanical properties of rat femurs was assessed by 3-point bending tests, and the mechanical properties of bone around the implants were assessed by removal torque test. Fresh femurs from the first animal study after microCT scanning were prepared for 3-point bending tests, and fresh femurs from the second animal study after microCT scanning was prepared for removal torque mechanical study. Load vs. displacement graph or load vs. radian graph was generated for each femur and fit to a bilinear model (**a**) to calculate max load (**b**), stiffness (**c**), and toughness (**d**) from a 3-point bending test, and max load (**e**), torsional stiffness (**f**), and yield point (**g**) were calculated from removal torque mechanical test. Data within each group not sharing a letter are significantly different (A vs. B vs. C vs. D for example) at an α = 0.05 by one-way ANOVA with Tukey hoc-post correction for multiple group comparison for graphs. Groups that share a letter (A vs. A or AB vs. B for example) are not significantly different (**b**–**d**). Two-way ANOVA was used to compare groups in graphs (**e**–**g**).

Mechanical analysis of hindlimbs by three-point bending (Figure 5a) showed no differences between vehicle and botox treatment with or without sema3A treatment for the maximum load (Figure 5b), stiffness (Figure 5c), and toughness (Figure 5d). Mechanical torque to failure was used to quantify the material properties of the newly formed bone around PT and SLAnano implants. In healthy rats, SLAnano implants robustly increased the maximum load (Figure 5e), torsional stiffness (elastic modulus) (Figure 5f), and yield point (Figure 5g), which was consistent with the higher amount of trabecular bone observed in representative microCT images (Figure 4a,g). Compared to PT, the use of SLAnano in BTX-compromised rats increased the integrated bone mechanical properties more than three-fold (Table S1). Botox injections reduced the maximum load, torsional stiffness, and yield point for rough titanium implants regardless of sema3A treatment (56% reduction) (Figure 5e–g). At the same time, there was no difference in the mechanical properties of bone integrated into PT implants when comparing botox-injected rats with healthy rats (Figure 5e–g). Additionally, sema3A did not affect the mechanical properties of bone attached to either type of implant. Overall, our data showed that titanium implants with a biomimetic surface topography demonstrated the clinical advantages of increasing osseointegration for mechanically unloaded situations compared to smooth titanium implants. The addition of sema3A increased the amount of bone attached to the implants while not affecting the mechanical properties.

## 4. Discussion

This study confirmed our previous observations that mechanically unloaded bone in botox-treated rat femurs exhibits a compromised phenotype characterized by reduced trabeculae in the metaphysis and reduced cortical bone in the diaphysis. Consistent with our previous study [40], we did not observe any botox-related toxicity issues in terms of disrupting normal eating, high-stress levels, or other concerns. The results of the present study showed that botox treatment reduced the mechanical stability of transcortical Ti implants. The effect was greatest for implants that lacked a biomimetic surface topography. Our results also show that treatment with sema3A via injection did not mitigate the effects of mechanical unloading resulting from botox injection. However, if sema3A was delivered to the treatment site in a biodegradable Cu-free click hydrogel, it was able to mitigate the impact of botox. Interestingly, this ability to overcome the negative impact of botox was limited to sites receiving implants with biomimetic surface topography.

Pathologies, where the muscle function is chronically disrupted, have been proven to affect skeletal health [45,46,47]. In conditions such as bed rest and spinal cord injury, bone loss is rapid and acute, ranging from 5% to 25%, depending on the skeletal sites and injury severity [48]. Rodent models representing these clinically mechanically unloaded or muscle disuse conditions, including tail suspension, cast immobilization, intramuscular botox injections, and tendon resection, exhibit bone loss, which might potentially jeopardize bone regeneration [49,50,51,52].

Our results showed that botox injections dramatically decreased trabecular and cortical bone in femurs at three different locations: the distal metaphysis, the mid-shaft, and the proximal side. The muscle paralysis induced by botox injections compromised the whole bone phenotype, as noted previously [53,54,55], whereas the contralateral legs were unaffected. Our previous work also showed that not only was the bone phenotype affected by botox injections, but the regenerative ability of bone tissues with implants that had a hydrophilic biomimetic topography was also impaired to a greater extent compared to neurectomy [40].

Osseointegration is a complex biological event consisting of stem cell recruitment, primary bone formation, bone remodeling, and mature bone formation [56]. Improvements in osseointegration can be approached by improving net bone formation during primary bone formation or by balancing bone formation and bone resorption during the remodeling phase, both of which are interrupted by diseases such as osteoporosis and diabetes [57,58,59,60].

In the current study, a nerve-derived factor, sema3A, was used to evaluate its therapeutic potential in improving bone formation. Sema3A has been shown to increase osteoblastic differentiation, inhibit osteoclast resorption in vitro, and improve bone formation in animal models, including osteoporotic rabbits and mice and diabetic rats [61,62,63]. Our data indicate that sema3A successfully improves botox-induced cortical bone loss to a similar level as the healthy rats with only two burst releases to the periosteum of the third trochanter.

Our data also showed that the effect of sema3A was extremely localized. This may have been due to the limited residency of sema3A after the injection of the periosteum. We did not test this clinically important question in the present study. If the effect of sema3A is limited to the injection site, it can be used locally in areas of regeneration without affecting the bone distal to the area of regeneration.

We used a rapidly polymerizing Cu-free click chemistry hydrogel to achieve local sema3A delivery to the implant insertion site. We have used this hydrogel to deliver a variety of factors to bone sites without observing any evidence of toxicity to the surrounding tissues [43,44]. The use of our Cu-free click hydrogel for the delivery of sema3A into rat bone sites was investigated in our previous study, including sema3A release kinetics [41]. The rapid in situ polymerization of the hydrogel demonstrated minimal swelling and remained cohesively intact under physiological conditions [41,43,44]. As anticipated, sema3A delivered via the hydrogel was biologically active and restored bone-to-implant contact in the cortical region where the factor was injected. It was retained locally and did not affect BIC in the marrow cavity. Importantly, its effect was evident only when the implant had a biomimetic surface topography.

Previous studies have demonstrated that making use of the Ti implants’ physical surface features can encourage peri-implant bone growth and osseointegration in several challenging conditions [12,13,14,15,42]. The roughness produced by the grit-blasting and acid-etching processes results in craters varying from 30 to 100 μm, overlaid with pits in the range of 1 to 3 μm. SLAnano has additional mesoscale and nanoscale features. This complex multi-scale topography contributes to better bone development and osseointegration by mimicking the natural structure of osteoclast resorption pits on the normal bone surface [8,64]. Here, we investigated the contributions of surface topography for improving osseointegration in this compromised model and showed that implants with a microscale/mesoscale/nanoscale structure significantly improved regenerated bone quality by improving the maximum load the bone can bear before failure, increasing the recovery ability of the bones at certain loads, and the higher endurance of loads before permanent damage occurs. The biomimetic surface topography did not overcome the negative botox effect on the mechanical properties to any greater extent than we observed previously with modSLA implants, which had the same topography but were hydrophilic rather than hydrophobic [40]. However, our data do indicate that biomimetic surface topography contributes to the success of additive approaches, potentially by providing an osteogenic microenvironment that can be further enhanced pharmacologically [28].

The observation that sema3A treatment further enhanced BIC on multiscale biomimetic surfaces but not on smooth surfaces was consistent with in vitro observations [28]. Sema3A increased the production of osteoprotegerin by MSCs cultured on Ti surfaces with a biomimetic topography. Sema3A is a coupling factor that can increase bone formation and decrease bone resorption [29] and osteoprotegerin is a decoy factor that can inhibit osteoclast differentiation [65]. These observations suggest that sema3A increased BIC by increasing bone formation or decreasing bone resorption, achieving net bone formation. The present data also suggest that the osteogenic factors generated by cells on biomimetic multiscale topographies work in concert with exogenous sema3A, whereas cells on smooth surfaces either do not produce these factors or produce them at concentrations that are not sufficient for a synergistic effect, especially in compromised bone conditions.

The use of botox in this study was to create a mechanically unloaded situation that mimics clinical conditions, such as patients with neuromuscular injuries and spinal cord injuries or are recovering from prolonged bed rest or long-term use of wheelchairs, as well as patients who have experienced microgravity. The negative impact of botox on the osseointegration of Ti implants was greater than observed in a neurectomy model that also mechanically unleaded the femoral bone [40]. However, our findings show that data impaired osseointegration could be improved by surface modifications to mimic the natural bone environment in combination with sema3A, although sema3A did not show the advantages of improving either the whole bone mechanical properties or mechanical properties of regenerated bone around implants at the periods we checked, even though our data showed that sema3A increased bone formation and BIC. The results of the present study also might indicate a therapeutic method to improve osseointegration in patients with compromised bone regeneration. Further studies emphasizing the concentration and delivery of sema3A can be modified for clinical use to improve osseointegration more than rescue.

## 5. Conclusions

The biomimetic concept of providing surface multiscale topography to resemble the natural bone structure is a promising tool for enhancing osseointegration in compromised bone-like disuse conditions, especially when surface modifications are combined with local factors produced by surface-cultured osteoblastic lineage cells. Titanium implant surfaces with a multiscale micro/nano texture exhibited the advantage of increasing the mechanical properties of integrated bone in both healthy and botox-compromised rats. Moreover, with the addition of sema3A, the deleterious effect of botox on osseointegration was restored to healthy levels. Clinically, it is important to understand the detrimental effect of disuse on the bone morphology and success rates of Ti implants. The findings have inspired clinicians to consider biomimetic approaches involving surface modifications and biologics combinations for improving implant outcomes in compromised patients.

## Data Availability

The data that support the findings of this study are available from the corresponding authors by request.

## Supplementary Materials

The following supporting information can be downloaded at: https://www.mdpi.com/article/10.3390/biomimetics8010093/s1, Figure S1: The evaluation of trabecular bone phenotype at the metaphysis of distal femurs from the first animal study; Figure S2: The evaluation of cortical bone phenotype at the semaphorin 3A injection sites; Table S1: Evaluation of osseointegration by microCT and removal torque testing.
