# Supplementary material for: Osseointegration of Titanium Implants in a Botox-Induced Muscle Paralysis Rat Model Is Sensitive to Surface Topography and Semaphorin 3A Treatment
Source: Biomimetics (Basel). 2023 Feb 25;8(1):93. doi: 10.3390/biomimetics8010093 (PMC10046785; doi:10.3390/biomimetics8010093)
Supplement: Supplementary file 1 [file biomimetics-08-00093-s001.zip › Supplemental Figure Legends.pdf]

Supplemental Figure 1. The evaluation of trabecular bone phenotype at the metaphysis of distal femurs from the first animal study. Femurs were harvested and placed in 1XPBS for microCT scanning. The trabecular bone phenotype at the metaphysis of the right hindlimbs with treatments was assessed by Bone Volume/Total Volume (a), Trabecular Thickness (b), Trabecular Number (c), and Total Porosity (d). The trabecular bone phenotype of all hindlimbs from all groups was assessed by Bone Volume/Total Volume (e), Trabecular Thickness (f), Trabecular Number (g), and Total Porosity (h). Data were shown as mean for each group ± standard error of n=5 for the veh group, n=8 for the veh+Sema3A group, n=8 for the BTX group, and n=9 for the BTX+Sema3A group. Groups not sharing a letter were significantly different by one-way ANOVA with Tukey hoc-post correction (α =0.05).

Supplemental Figure 2. The evaluation of cortical bone phenotype at the semaphorin 3A injection sites. Femurs were harvested and placed in 1XPBS for microCT scanning. The cortical bone phenotype at the semaphorin 3A injection sites of the right hindlimbs with treatments was assessed by Bone Volume/Total Volume (a), Total Porosity (b), and Cortical Thickness (c). The cortical bone phenotype at the sema3A injection sites of all hindlimbs from all groups was assessed by Bone Volume/Total Volume (e), Total Porosity (e), and Cortical Thickness (f). Data were shown as mean for each group ± standard error of n=5 for the veh group, n=8 for the veh+Sema3A group, n=8 for the BTX group, and n=9 for the BTX+Sema3A group. Groups not sharing a letter were significantly different by one-way ANOVA with Tukey hoc-post correction (α=0.05).
