# Supplementary material for: Osseointegration of Titanium Implants in a Botox-Induced Muscle Paralysis Rat Model Is Sensitive to Surface Topography and Semaphorin 3A Treatment
Source: Biomimetics (Basel). 2023 Feb 25;8(1):93. doi: 10.3390/biomimetics8010093 (PMC10046785; doi:10.3390/biomimetics8010093)
Supplement: Supplementary file 1 [file biomimetics-08-00093-s001.zip › Table 1.pdf]

Implants	Group	Total BIC	Marrow BIC	Cortical BIC	Max Load	Torsional Stiffness	Yield Point
**SLAnano**	Control	33.00±1.61 A	22.95±1.87 A	61.57±3.06 A	0.16±0.01 A	1.92±0.09 A	0.16±0.02 A
	BTX	22.02±2.86 B	18.22±2.96 A	35.70±3.83 B	0.07±0.01 B	1.33±0.14 B	0.05±0.00 B
	BTX+ Sema3A	29.36±2.93 AB	25.51±3.30 A	47.20±1.56 C	0.08±0.01 B	1.33±0.15 B	0.07±0.01 B
**PT**	Control	38.42±4.02 A	34.02±4.26 A	41.21±6.76 A	0.04±0.01 A	0.60±0.11 A	0.02±0.00 A
	BTX	18.27±2.11 B	17.04±2.38 B	30.31±6.79 A	0.02±0.00 B	0.31±0.09 A	0.01±0.00 B
	BTX+ Sema3A	20.54±1.64 B	19.75±1.82 B	31.07±4.66 A	0.02±0.00 B	0.29±0.08 A	0.01±0.00 AB

Table 1. Evaluation of osseointegration by microCT and removal torque testing. Results presented as mean ± SEM. Groups not sharing letters are different at α=0.05 using one-way ANOVA with Tukey post correction. Abbreviations: BIC, bone-to-implant contact.
