# Supplementary figures and images for: Osseointegration of Titanium Implants in a Botox-Induced Muscle Paralysis Rat Model Is Sensitive to Surface Topography and Semaphorin 3A Treatment
Source: Biomimetics (Basel). 2023 Feb 25;8(1):93. doi: 10.3390/biomimetics8010093 (PMC10046785; doi:10.3390/biomimetics8010093)

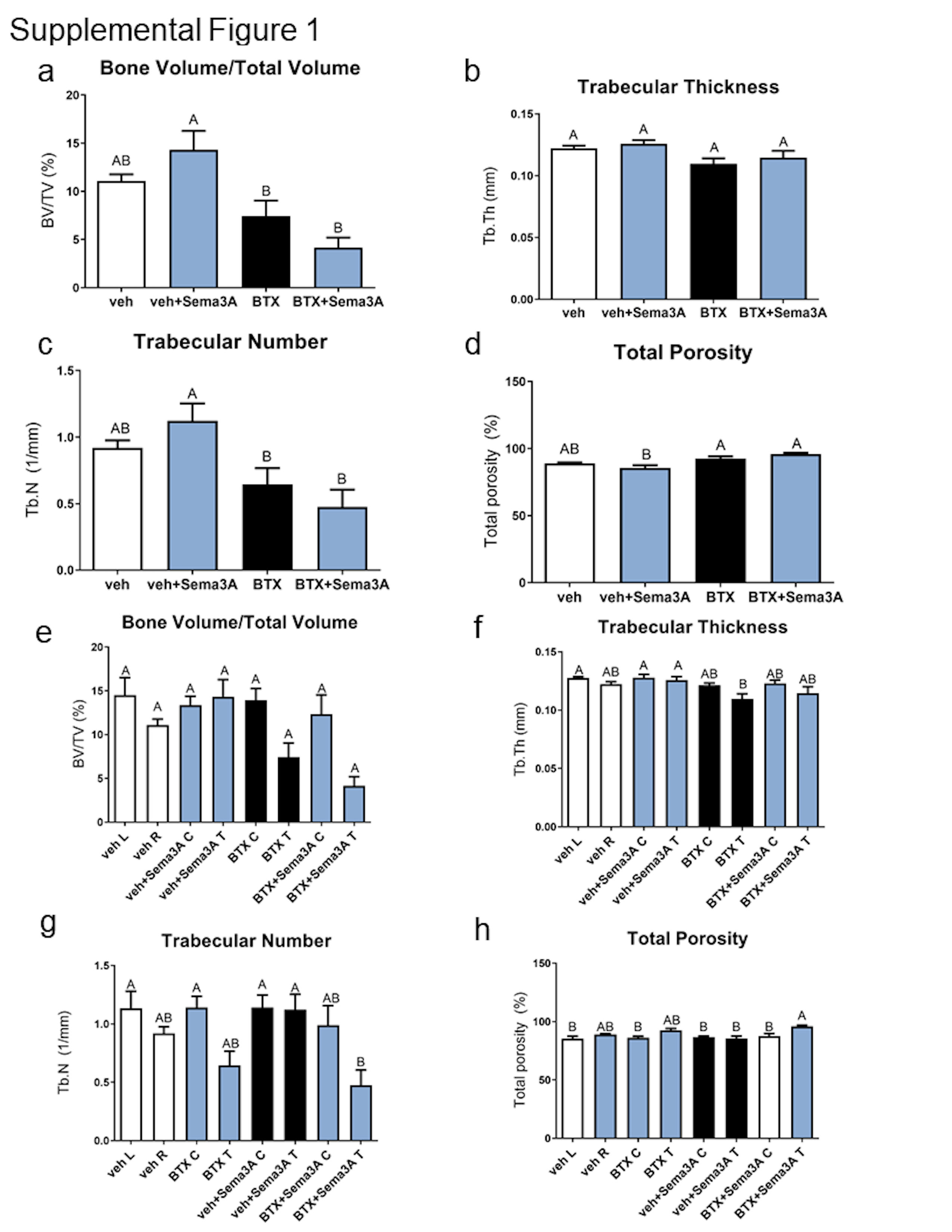

Supplement: Supplementary file 1 [file biomimetics-08-00093-s001.zip › Supplemental Figure 1.TIF]

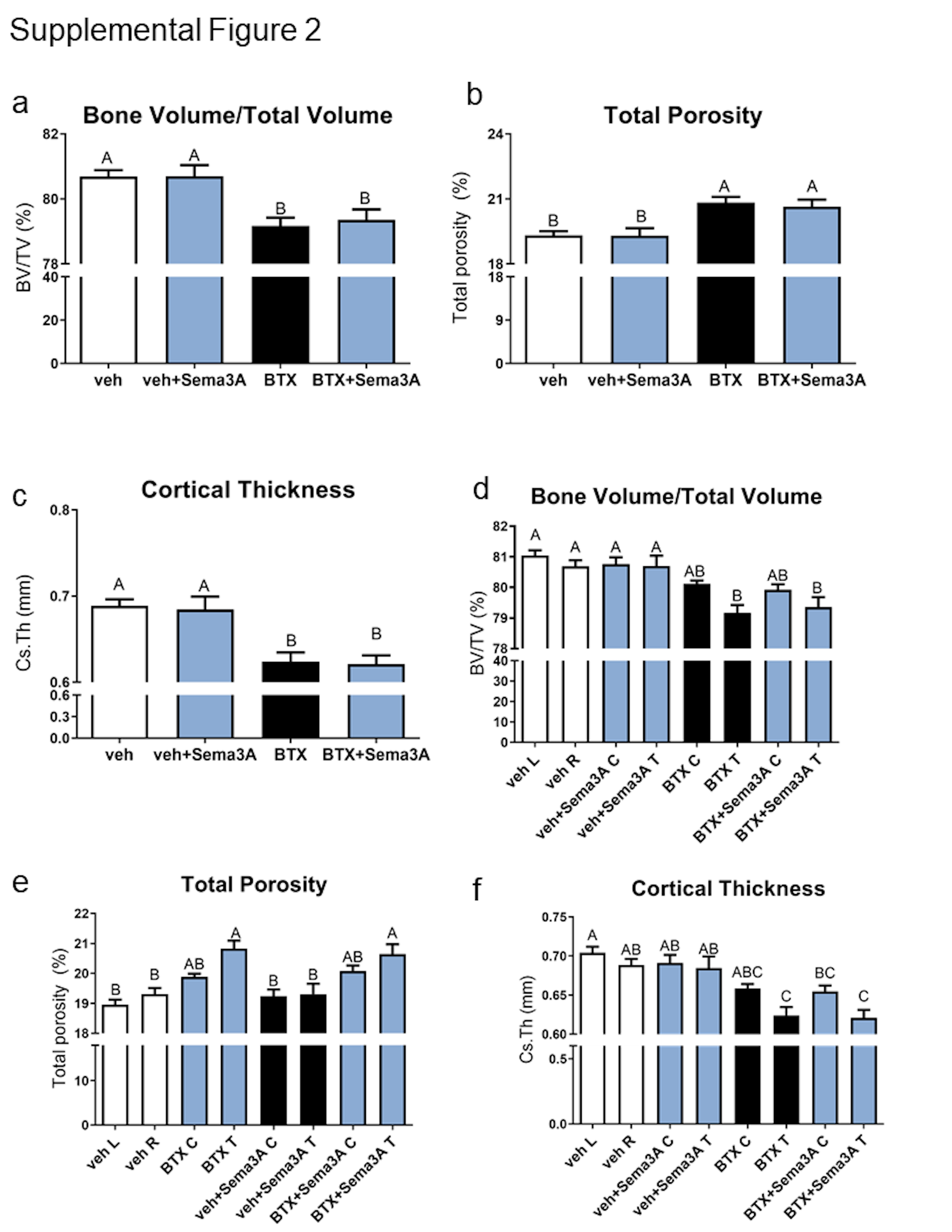

Supplement: Supplementary file 1 [file biomimetics-08-00093-s001.zip › Supplemental Figure 2.TIF]
